# Impacting on factors promoting intra-group aggression in secure psychiatric settings

**DOI:** 10.1016/j.heliyon.2019.e01400

**Published:** 2019-03-25

**Authors:** J.L. Ireland, I. Sebalo, K. McNeill, K. Murphy, G. Brewer, C.A. Ireland, S. Chu, M. Lewis, L. Greenwood, T. Nally

**Affiliations:** a University of Central Lancashire, Preston, UK; b Ashworth Research Centre, Mersey Care NHS Trust, UK; c University of Liverpool, UK

**Keywords:** Psychiatry, Clinical psychology, Psychology

## Abstract

Three preliminary and linked studies investigate the impact of making alterations to factors considered relevant to engaging in and experiencing intra-group aggression (bullying) among adult male patients detained in a single secure forensic hospital. Study one (n = 44) outlines the institutional factors, attitudes towards bullying and environmental factors that increase the likelihood of engaging in bullying and/or being victimised. Study two (n = 53 patients and 167 staff) assesses the effect of three variations of intervention that aimed to reduce intra-group aggression through direct alteration of the physical and psychosocial environment, using data from both patients and staff. Study three (n = 414) looks at the effects of two variations of the intervention used in study two, which offered patients’ participation in individual and communal activities. It was predicted that changes to the physical and social environment would produce a reduction in the factors shown to predict intra-group aggression. Attitudes supportive of bullying and the presence of social hierarchies each increased the likelihood of engaging in bullying. Indirect changes to the social environment on the wards had more positive effects than those incorporating direct alterations to the physical and social environment. The differences in effectiveness of the two approaches are discussed in relation to the established predictors of intra-group aggression. The research concludes by noting the preliminary nature of the research and outlining potential directions for future research and intervention.

## 1 Introduction

One of the main aims of forensic psychiatric care is to reduce the risk of violent conduct among patients (Andrews et al., 2006). Evidence for intra-group aggression (bullying) among patients clearly conflicts with this aim. As a topic of academic interest there has been little attention afforded to secure forensic psychiatric units on intra-group aggression. Research has, instead, focused on the concept of school bullying, where a narrow definition comprising of four elements tends to be applied; namely, that in order for a behaviour to be considered bullying it must include an intention to cause harm, be repeated behaviour, include physical or psychological aggression, and have a basis in unequal power between the parties involved (Farrington, 1993). This definition has been criticised as the area of research has evolved and extended beyond schools. In order to be applied in secure settings, the definition arguably requires some adjustment.

In a series of semi-structured interviews, Ireland (2005) explored perceptions of bullying among patients and staff members in a high secure hospital. Findings demonstrated that bullying can be accidental, can constitute a single incident, and is not necessarily rooted in power imbalance, with more than half of victims not perceiving assailants as holding more power than them. Another noteworthy aspect was that the term “bullying” itself was considered by participants likely to be associated exclusively with the behaviour of children. Consequently, using the term could lead to underreporting by older age groups when asked directly ‘have you bullied/been bullied?’ Indeed, the majority of patients (80%) and staff (63%) interpreted ‘bullying’ as a descriptor of children's behaviour. Consequently, it has been proposed that in secure settings bullying is best described as intra-group aggression, characterised by the *perception* of being victimised by others, and by a victim's *fear of the potential recurrence* of similar incidents, regardless of actual reality (Ireland, 2004, 2012). Thus, it can represent a single occurrence of direct (explicit) or indirect (subtle) aggression. Power imbalance is also discounted as not necessarily being clearly present.

Approaches that have adopted a more discrete behavioural means of measuring intra-group aggression, one that does not use the term ‘bullying’, have provided repeated indications across studies of a sizeable amount of aggression taking place between residents in secure settings. Using one such example, The Direct and Indirect Patient (Prisoner) behaviour Checklist (DIPC: Ireland, 1999; Ireland and Ireland, 2008; Ireland, 1999; Ireland and Bescoby, 2005), has demonstrated intra-group aggression to be routine for some. Separate weekly/monthly rates of bullying and victimisation in prisons and secure psychiatric hospitals rarely fall below 40% and can reach 80%, with indirect aggression (i.e. subtler) aggression often reported to be more prevalent (Ireland and Bescoby, 2005; Ireland and Rowley, 2007; Cooper et al., 2011; Haufle & Wolter, 2015; Ireland and Ireland, 2008). The research has been focused largely on men, however, and there is some evidence of increased proportions of aggression among younger than older groups but, nevertheless, it suggests that almost every second person within secure settings has either aggressed toward a peer or was aggressed against. More precisely, it is likely that a person has been both a bully *and* a victim, as the number of those belonging to the ‘bully/victim’ group is often higher than those who are purely victims, purely bullies, or are not involved (Ireland and Rowley, 2007; Ireland and Power, 2004; Sekol, 2016).

In order to facilitate an understanding of the causes of intra-group aggression in secure forensic psychiatric settings, Ireland and Snowden (2002) argued the behaviour to be a reflection of environmental *and* individual factors associated with such settings, drawing on prison based research. They proposed a rigid institutional structure based on dominance hierarchies, as important considerations, coupled with a patient subculture that condemned informing on others, high densities of individuals residing in limited space, a raised turnover of residents, lack of available activities and appropriate levels of (unpredictable) supervision as core environmental factors related to intra-group aggression. Social as well as physical factors were also deemed important, with individual beliefs supporting and justifying the use of aggression, demeaning perceptions of victims, and poor empathetic abilities highlighted as further facilitating bullying (Ireland and Snowden, 2002). In 2010 Allison and Ireland measured evidence for these factors using the Prison Environment Scale (PES), demonstrating a clear association between an increased prevalence of factors and intra-group aggression among prisoners (Allison and Ireland, 2010). Attitudes appeared as a particularly important factor and arguably interacted with other environmental factors. The presence of attitudes favouring bullying, such as lack of support for victims, perception of bullies as skilled, admiring bullies, and victim blaming, were positively associated with the institutional factors that supported intra-group aggression (Ireland et al., 2016). Similarly, Cooper et al. (2011) revealed an association between beliefs comprising Machismo cognition (characterised by a normalisation of aggression) and attitudes that supported bullying among patients detained in a medium secure hospital, suggesting that belief structures may be of particular value.

Uniting the known facilitators and causes of intra-group aggression in secure settings, Ireland (2012) proposed the *Multifactor Model of Bullying in Secure Settings* (MMBSS). The MMBSS was a revision of the previous *Interaction Model of Prison bullying* (IMP) proposed in 2002 (Ireland, 2002). The IMP was a simple model that argued how the social and physical aspects of a secure environment interacted with individual characteristics to promote bullying. The MMBSS proposed a more detailed understanding using pathways that accounted for individual factors being stable and/or dynamic and placed more emphasis on a role for attitudes. According to the MMBSS, the primary roots of intra-group aggression are located in the environment (Ireland, 2012). It presents a *desensitization pathway* that reflects how the raised frequency of acts of aggression and/or threat of the same in a hostile setting normalises and facilitates aggression related beliefs and attitudes. These, in turn, interact with acute emotional states, such as stress, fear and/or anger, to promote engagement in aggression. Distinct to this is the *environment and prior characteristic* pathway, which reflects how the physical and social surroundings enhance pre-existing aggression supportive traits, encouraging their expression. The MMBSS, like the IMP, divides the environment into physical and social. The former includes limitations on material goods, poor quality and low quantity of supervision (including raised predictability of supervision), and increased spatial and social density. The latter incorporates power-based dominance hierarchies, poor attachment relationships, and a subculture favouring aggression and encouraging disengagement from staff.

The MMBSS was partly confirmed in a large-scale study on intra-group aggression among adolescents in care (Sekol, 2016). It was found that the lack of peer support was the best predictor of both bullying and victimisation, highlighting the importance of social environment (community) related factors. Moreover, male bullies reported more experiences of unfair treatment from staff, concerns about physical aspects of the environment and general diminished wellbeing. Collectively these findings favoured a role for the environment as a notable correlate with bullying. The importance of social climate and the community in precipitating aggressive behaviour, as indicated in the MMBSS, was also confirmed by the findings of Ros et al. (2013). Based on data gathered from 72 patients in secure settings and prolonged intensive care, they identified a decrease in ward climate (characterised by patient's perception of limited possibilities for learning and growth) and diminished professional support as predictors of an increase in aggressive incidents. Interestingly, however, an atmosphere of trust between patients and perception of unjust system of rules on the wards, did not predict any change in the number of aggressive incidents. Ros et al. (2013) further demonstrated that a positive social climate on the wards, characterised by patients' safe interactions with one another and opportunities for personal development, was likely to occur when aggressive incidents were not present.

The MMBSS, however, remains a theoretical model that requires further exploration and empirical testing. This is not unique to the MMBSS, however, since there remains no research to date that has empirically assessed interventions aimed at preventing intra-group aggression/bullying in secure services, including psychiatric settings (Ireland et al., 2016). The MMBSS does, nevertheless, provide some indication of what could be considered a theoretically informed means of intervening positively into intra-group aggression. For example, according to the MMBSS, enhancing the social environment in secure settings should have a detrimental effect on bullying. Creating a sense of community among patients presents as one example of a potentially salient factor to consider.

In community settings, it is well recognised that poor community identification is associated with antisocial coping, negative mood and low social joining (Roussi et al., 2006). In secure settings, victims of intra-group aggression have reported raised levels of emotional loneliness (Ireland and Power, 2004) and social isolation (Connell et al., 2016), suggesting a poor connection with others and yet a desire to belong. Traditionally, a positive sense of community includes four main components; membership; emotional connection with other members; ability to influence the community; and being able to meet individual needs with the help of the community (Chavis et al., 2008). Conversely, a *negative* sense of community has four components that facilitate individual disengagement (Mannarini et al., 2014); Frustration, which can arise from an inability to fulfil one's needs through a community; distinctiveness, which refers to an active rejection of a community based on one's view of being different; abstention, reflecting a passive position that facilitates avoidance of engaging with a community; and alienation, to denote a sense of community alienation.

Given the relevance that the MMBSS attributes to the social climate, it is surprising that interventions aimed at increasing a sense of community have not been implemented in secure settings as a potential remedy to intra-group aggression. The current research addresses the lack of attention given to this area through a series of connected studies, all of which take place with adult male forensic psychiatric patients who are detained in conditions of high security in the same hospital. It commences by exploring elements of the MMBSS that could relate to bullying and/or victimisation (Study 1), before moving onto to examine the impact of making specific changes to the environment as a means of positively impacting on intra-group aggression (bullying and/or victimisation). Based on the MMBSS, the following predictions were made: 1.) Physical environmental factors associated with intra-group aggression will predict engagement in bullying and of being victimised; 2.) Social environmental factors, namely attitudes supportive of bullying, will predict engagement in bullying and of being victimised; 3.) Making positive changes to aggression-enhancing aspects of the physical and/or social environment will individually reduce the factors associated with intra-group aggression and experiences of the same; 4.) Making positive changes to aggression-enhancing aspects of the physical and social environment together will produce a greater reduction in the factors associated with intra-group aggression and experiences of the same than just a focus on either the social or physical environment; 5.) Enhancing a sense of community will positively impact on the factors associated with increasing intra-group aggression.

## 2 Materials and methods

### 2.1 Study one

This exploratory study examines the predictors and correlates of intra-group aggression, which arise from the psychosocial and physical environment, in a high secure psychiatric setting. The individual predictors established will subsequently be used in the ensuing studies as indicators of bullying and victimisation to assist with evaluation.

#### 2.1.1 Participants

Forty-four adult male patients from a high secure hospital in the UK took part. The age of participants ranged from 21 to 56 (with most, 53%, aged between 21 and 31). Eighty-four per cent described themselves as White British. Twenty-seven per cent were classified under the Mental Health Act (MHA) as having mental illness, 23% as having psychopathic disorder, and 50% as having psychopathic disorder and mental illness. Time spent in a hospital ranged from seven to 396 months (M = 70, SD = 72.9).

#### 2.1.2 Measures

*Direct and Indirect Patient behaviour Checklist-Hospital version Revised (DIPC-HR: see* Ireland and Rowley, 2007*),* a self-report behavioural checklist with yes/no answers assessing direct physical, verbal, sexual and psychological bullying. Within the checklist are 138 aggression items, split equally between assessing perpetration and victimisation. It does not use the term ‘bullying’. Rather, items ask about discrete behaviours, e.g. ‘Someone has deliberately started a fight with me’, ‘I have deliberately humiliated someone’.

*Patient/Prisoner Bullying Scale (PBS: see* Ireland et al., 2009*)* to measure attitudes supportive of bullying. This 39 item Likert scale self-report questionnaire has six attitude dimensions; negative and blaming attitudes towards victims (e.g. ‘Patients should be able to dominate others and get away with it’); believing that bullying can have positive connotations (e.g. ‘Patients who are victimised usually enjoy getting bullied’); supporting victims and disapproving of bullying (e.g. ‘Patients who are weaker than others should be helped’); seeing victims as attention seeking (e.g. ‘Patients only report bullying to get attention from staff’); perceiving bullies as skilled (e.g. ‘Bullies are physically stronger than other patients’); and protecting victims (e.g. ‘Patients should never pick on someone who is weaker than them’). The PBS has been found to be internally reliable (e.g. minimum α = .81; Ireland et al., 2009).

*Prison Environment Scale (PES:* Allison and Ireland, 2010*)* to measure the institutional factors that facilitate bullying. This 40 item Likert-scale self-report questionnaire estimates the presence of the physical and social factors that facilitate bullying. It was adapted here for use in a secure hospital. The social factors are based on the MMBSS and incorporate existence of hierarchy and order, beliefs that bullying is inevitable, and power and organisational structures. Physical factors include items relating to an absence of meaningful activities, raised social density and predictable supervision. Example items include: ‘There are no activities to keep patients occupied’, ‘There is a high turnover of patients’, and ‘Patients always know when staff will be present’. The PES has been found to have moderate reliability (α = .70; Allison and Ireland, 2010).

*Essen Climate Evaluation Schema (EssenCES:* Schalast et al., 2008*)* was employed to assess how patients and staff members view the ward atmosphere. It is a 15 item Likert-scale assessment capturing patient cohesion (e.g. ‘The patients care for each other’), experienced safety (‘Some patients are afraid of other patients’), and therapeutic hold (‘Staff take a personal interest in the progress of patients’). Internal reliability for a similar sample ranged from moderate to good (α .72 to .92; Tonkin et al., 2012).

#### 2.1.3 Procedure

Patients were approached with a questionnaire pack to complete on their own. Participation was voluntary and they were informed that they could withdraw at any time. All questionnaires were anonymous. All analysis was conducted using SPSS vs. 24.0. All participants consented and ethical approval was obtained from the University of Central Lancashire Ethics Board (Psychology) and regulations complied with.

## 3 Results

### 3.1 Study one

Taking into account a relatively small sample size and in order to reduce the number of tests applied, correlational analysis was initially conducted to identify the most likely predictors of being a bully and a victim, among the attitudes towards bullying, institutional factors, and ward atmosphere. In order to establish the potential predictors for behaviours associated with total bullying and victimisation, Spearman's test^1^ was run between all subscales of the PBS, PES, EssenCES and total scores for bullying and victimisation on the DIPC-HR (Ireland and Rowley, 2007). Table 1 presents the results.

**Table 1:** Spearman correlations between attitudes supporting bullying, institutional factors, ward atmosphere and engaging in bullying and experiencing victimisation (n = 44).

	Total bullying behaviour		Total victimisation	
	Rho	Sig	Rho	Sig
**PBS total score**	**.33**	**.03**	.19	.23
Negative and blaming attitudes towards victims	**.34**	**.03**	.23	.13
Belief that bullying can have positive connotations	**.32**	**.04**	.12	.45
Supporting victims and disapproving of bullying	−.02	.91	.02	.92
Seeing victims as attention seeking	**.36**	**.02**	.03	.83
Perceiving bullies as skilled	.22	.16	.04	.83
Victim protecting attitudes	.11	.47	.06	.682
**PES total score**	**.42**	**.005**	**.38**	**.01**
Existence of hierarchy and order	**.48**	**.001**	**.35**	**.02**
Belief that bullying is inevitable	**.36**	**.02**	.22	.15
Absence of meaningful activities	**.30**	**.049**	.28	.07
Raised social density	**.32**	**.04**	**.46**	**.002**
Predictable supervision	.29	.06*	.15	.33
**EssenCES total score**	−.13	.4	−.2	.21
Patient cohesion	.02	.91	.04	.79
Therapeutic hold	.10	.54	−.03	.86
Experienced safety	−.29	.06*	−**.34**	**.03**

*Note*. Values in bold are significant; * <.10.

Results demonstrated that total PBS and PES scores were associated with increased reports of bullying perpetration, with the PES also associated with increased reports of being victimised. Some subcomponent elements of both the PES and PBS related to bullying perpetration, with some PES subscales also relating to victimisation. Meanwhile, experienced safety, from the EssenCES, was the only variable to correlate with victimisation, presenting with a low negative association.

On the basis of this, separate binominal regressions were then employed (Table 2) to assess the individual predictive power of the institutional factors, attitudes supportive of bullying, and ward atmosphere identified (Table 1) as associated with bullying or victimisation.

**Table 2:** Predictors of engaging in bullying and experiencing victimisation (n = 44).

Category	Predictor	Exp. B	S.E.	Sign.	Model Chi square	Hosmer Lemeshow test	Nagelkerke R^2^	% Correctly classified	2 Log Likelihood
**Victimisation**	**PES total**	**1.05**	**.02**	**.02**	**10.3**	**.24**	**.23**	**72**	**47.76**
Existence of hierarchy and order		**1.11**	**.05**	**.03**	**8.86**	**.35**	**.18**	**69.8**	**49.64**
Raised social density		**1.36**	**.14**	**.02**	**6.32**	**.28**	**.19**	**67.4**	**49.19**
Experienced safety (EssenCES)		.89	.07	.12	13.41	.10	.09	69.8	52.82
**Bullying**	**PBS total**	1.02	.01	.8	5.84	.67	.14	69.8	51.02
Negative and blaming attitudes towards victims		1.08	.05	.12	9.05	.11	.11	65.1	51.92
Belief that bullying can have positive connotations		**1.12**	**.05**	**.04**	**3.15**	**.68**	**.16**	**69.8**	**50.2**
Seeing victims as attention seeking		1.16	.1	.13	1.78	.62	.08	67.4	53.23
	**PES**	**1.06**	**.02**	**.01**	**10.4**	**.24**	**.28**	**76.7**	**45.83**
Existence of hierarchy and order		**1.16**	**.05**	**.005**	**16.68**	**.04**	**.33**	**81.4**	**43.97**
Belief that bullying is inevitable		**1.39**	**.15**	**.02**	**6.87**	**.33**	**.19**	**74.4**	**49.2**
Absence of meaningful activities		1.29	.14	.08	6.91	.44	12	67.4	52.18
Raised social density		1.33	.15	.06	2.66	.75	.14	65.1	50.9

*Note.* Values in bold were found to be significant.

Significant predictors were then subjected to ROC curve analysis to verify the correctness of the classification. For victimisation, total score on the PES was found to have a good AUC value: .73 (SE = .08, Asymptotic significance = .02, CI = .56-.89). Similarly, presence of hierarchy and order, and raised social density were found to have AUC: .72 and AUC: .74, respectively (SE = .09, Asymptotic significance = .02, CI = .55-.89; SE = .08, Asymptotic significance = .01, CI = .58-.9). Meanwhile, for engaging in intra-group aggression (bullying), the positive attitudes towards bullying had a poor AUC value of .65 (SE = .1, Asymptotic significance = .12, CI = 46-.84). However, total score on the PES had a good AUC = .76 (SE = .08, Asymptotic significance = .006, CI = .6-.91). Furthermore, the subscales of perceived inevitability of bullying and presence of hierarchy and order had good AUC values (AUC = .7 SE = .09, Asymptotic significance = .03, CI = .53-.87; and AUC = .8, SE = .07, Asymptotic significance = .001, CI = .66-.94, respectively).

## 4 Discussion

### 4.1 Study one

Attitudes towards bullying and institutional factors appeared particularly important considerations in identifying those associated with intra-group aggression. Although experienced perceived safety, a component of ward atmosphere, had a moderate negative association with victimisation, it was not identified as a predictor. Arguably this finding corresponds to that of Ros et al. (2013) who demonstrated that ward atmosphere was related to, but not predictive of, aggressive incidents. It would appear though that patients who have experienced more institutional factors associated with intra-group aggression were more likely to engage in bullying and to report victimisation by others. This is consistent with previous research demonstrating that those engaging in and suffering from intra-group aggression report higher levels of perceived institutional factors (Allison and Ireland, 2010). Regarding individual factors, only presence of rigid social hierarchy and order was predictive of both engaging in bullying and experiencing victimisation. However, the model including the perpetration of intra-group aggression had a significant Hosmer Lemeshow test, suggesting that it did not fit the data well.

In line with expectations, raised social density (one of the institutional factors) was found to be most predictive of victimisation, as those experiencing this have almost a 40% higher chance of being victimised by other patients. Since this dimension reflects an increase in the amount of individuals encountered during the day, these results suggest, somewhat logically, that exposure to a raised number of patients increases the chances of a given patient to being victimised. Interestingly, attitudes towards bullying were only predictive of engaging in intra-group aggression but not of being victimised, and even then the specific attitude was essential to account for. Specifically, the belief that bullying was inevitable was found to be the best predictor, with a reporting of such a belief raising the chances of engaging in the corresponding behaviour by almost 40%. This result is consistent with the desensitisation pathway proposed by the MMBSS since it is quite possible that this cognition is used as a precursor that facilitates perpetrating intra-group aggression at the start, and allows for its justification following.

The current study is not without its limitations. It is an exploratory small-scale study that is making inferences from the data. Use of a convenience sample indicates that such inferences must be made with caution. Nevertheless, these preliminary findings do converge with expectations from the literature and suggest value in using the institutional and attitudinal factors as constructs associated with intra-group aggression. Consequently, they are incorporated into the next component of the research programme, as a means of evaluating the effects of interventions designed to reduce intra-group aggression.

## 5 Materials and methods

### 5.1 Study two

Building on the earlier study, the research proceeded to evaluate implementation of an initial intervention that directly targeted the factors associated with intra-group aggression (bullying). Alterations to the environment were made on three wards in a high secure psychiatric (male) hospital and compared to a control (no-alteration) ward. The wards were selected by hospital management and not the researchers, to assist with our independence to the process. To explore what elements of the intervention may be impacting, there were three variations of intervention:

a.) Physical ward changes only;
b.) Social ward changes only;
c.) Combined physical and social ward changes.

Changes were guided by the proposed pathways of the MMBSS. Implementation also followed the recommendation of Smith et al. (2005), namely that it was preceded by a process of consultation with patients and staff regarding the proposed changes and comprised those that they felt would improve atmosphere on the ward.

As a result, specific improvements made to the physical surroundings entailed refurbishing (including improved area lighting) and redecorating (using patient art), an increase in the visible materials available for free time-activities (e.g. sport equipment, games and recreational supplies). In addition, staff members were tasked with promoting responsibility for the ward maintenance among the patient group.

Alterations to the social environment included raised opportunity for group-based activities. It also included ward group interactive sessions on anti-bullying awareness and interpersonal skills’ group sessions, delivered by therapists external to the ward. All patients on the ward were able to engage.

#### 5.1.1 Participants

Fifty-six patients and 113 staff members from four different wards were approached to participate; 33 (59%) patients and 98 (87%) staff agreed to participate in the first round of data collection. From these, 10 patients and 26 staff were from the ward that received no changes, 10 patients and 17 staff members were from the ward that received both types of changes, six patients and 26 staff from wards with only social changes, and seven patients and 29 staff members from the ward with only physical changes.

Twenty patients and 69 staff completed evaluations at the second time point (after the intervention), after a time period of four months. From these there were seven patients and 20 staff from the ward with no changes, five patients and 14 staff members from the ward with combined changes, five patients and 16 staff members from the ward with only social changes, and three patients and 19 staff members from the ward with only physical changes. The estimated values were used for the missing cases at the second time point, so those participants who only took part in the first round of data collection were not excluded.

Patient ages ranged from 21 to 56 (with most, 65%, aged between 21 and 41). Seventy-six per cent described themselves as White British. Sixty-four per cent were classified under the Mental Health Act (MHA) as having mental illness, 6% as having psychopathic disorder and 24% as having psychopathic disorder and mental illness. Six per cent chose not to disclose their diagnosis. Time spent in the hospital ranged from three to 180 months (*M* = 53.1, SD = 44.2) and on the identified ward from one to 168 months (*M* = 37.1, SD = 35.5). Staff ages ranged from 21 to 61+ (with most, 71.1%, aged between 42 and 56). Time spent working in the hospital ranged from 18 to 408 months (*M* = 204.2, SD = 99.9), with time spend on the current ward ranging from one to 168 months (*M* = 37.1, *SD* = 35.5).

#### 5.1.2 Evaluation measures

These comprised the Patient Bullying Scale (PBS), Prison Environment Scale (PES), and Direct and Indirect Patient behaviour Checklist-Hospital version Revised (DIPC-HR). These are outlined in Study 1. The DIPC-HR was only given to patients. All participants consented to complete the measures, with ethical approval again obtained from the University of Central Lancashire Ethics Board for Psychology, with regulations complied with.

## 6 Results

### 6.1 Study two

Due to the nested nature of the data, attrition rate and significant variance between (as well as within), mixed regressions were used to investigate the effects of the interventions. Initially, for each outcome variable a repeated measures model with an unspecified 2 × 2 covariance matrix for an individual effect at each time point was created, then a random within subject effect was added to the model (the Likelihood ratio (LR) test with *df* = 1 was used to determine fit of the new model). The predictors were time (pre and post intervention), group (dummy variables were created for combined, social and environmental intervention groups, while the control group was the reference point), and the interactions of each group with time. The differences between groups at the pre-intervention point, as well as the difference within the control group with the passing of time, were removed if the differences between models were not significant. The final model was also tested against the model without interactions, in cases when they were significant. Lastly, to account for the sample consisting of both staff members and patients, a Welch test was used to assess potential differences between these two groups in scores on subscales that significantly changed.

The following mixed effect regression model was used for each outcome score Y as obtained at the time *t* for an individual *i:*

Y_ti_ = β_0_ + β_1_PrePost_1_ + β_2_Combined_Changes + β_3_Social_Changes + β_4_Physical_Changes + β_5_PrePost*Combined_Changes + β_6_PrePost*Social_Changes + β_7_PrePost*Physical_Changes + *u*_*i*_ + *e*_*ti.*_

where t = 0 or 1 for baseline and post test respectively and post = 1 if t = 1,

*u*_*i*_ is a random effect of an individual *i*, and *e*_*ti*_ is a random effect of an individual *i* at the time point *t* also referred to as residual variance per individual per time point.
β_0_ - the outcome mean at the baseline in comparison group.
β_1_ – the mean change from baseline to post-test within the comparison group of patients.
β_2_ - the mean baseline difference between combined group of patients and comparison group.
β_3_ - the mean baseline difference between social group of patients and comparison group.
β_4_ - the mean baseline difference between environmental group of patients and comparison group.
β_5_ – the difference in mean change from baseline to post-test between combined and comparison group, which is also the difference at post-test, as there is no difference at baseline.
β_6_ - the difference in mean change from baseline to post-test between social and comparison group, which is also the difference at post-test, as there is no difference at baseline.
β_7_ - the difference in mean change from baseline to post-test between environmental and comparison group, which is also the difference at post-test, as there is no difference at baseline.

Tables 3 and 4 present the mixed regression analyses for attitudes and institutional factors respectively.

**Table 3:** Mixed regression analysis for attitudes supportive of bullying (n= 220).

		Estimate (S.E)	Sig	95 % CI		WS	Sig	BS	Sig
PBS Total	Combined ward	2.34 (3.10)	.56	−5.47	10.14	197.10 (34.39)	<.001	144.19 (46.24)	.002
	Social ward	−3.59 (3.76)	.34	−11.03	3.86				
	Environmental Ward	−1.93 (3.68)	.60	−9.22	5.35				
Negative and blaming attitudes towards victims	Combined ward	**2.40 (1.08)**	**.026**	**.26**	**4.53**	15.54 (2.52)	<.001	8.00 (2.92)	.006
	Social ward	−.94 (1.03)	.36	−2.98	1.10				
	Environmental ward	−.60 (1.01)	.55	−2.59	1.39				
Belief that bullying can have positive connotations^a^	Combined ward	−4.07 (2.31)	.08	−8.67	.53	16.62 (2.06)^c^	<.001	103.15 (15.86)^c^	.005
	Social ward	−1.61 (2.20)	.47	−5.98	2.77				
	Environmental ward	.34 (2.15)	.88	−3.94	4.62				
Supporting victims and disapproving of bullying	Time	−**2.16 (1.03)**	**.04**	−**4.20**	−**.13**	18.55 (2.83)	<.001	13.59 (3.6)	<.001
	Combined ward	**3.46 (1.54)**	**.03**	**.42**	**6.5**				
	Social ward	**4.07 (1.49)**	**.007**	**1.12**	**7.02**				
	Environmental ward	2.31 (1.48)	.12	−.61	5.22				
Seeing victims as attention seeking^b^	Combined ward	.08 (.58)	.89	−1.08	1.24	9.18 (1.14)^c^	<.001	6.57 (1.02)^c^	<.001
	Social ward	−.53 (.56)	.35	−1.63	.58				
	Environmental ward	.01 (.55)	.98	−1.07	1.10				
Perception of bullies as skilled	Combined ward	.66 (.97)	.5	−1.26	2.58	12.86 (2.01)	<.001	5.60 (2.18)	.02
	Social ward	−1.5 (.97)	.11	−3.33	.33				
	Environmental ward	−1.43 (.91)	.12	−3.22	.37				
Protecting victims	Social ward pre	**1.32 (.54)**	**.02**	**.25**	**2.40**	5.65 (.86)	<.001	1.83 (.84)	.03
	Combined ward	.52 (.64)	.42	−.74	1.77				
	Social ward	−.77 (.70)	.27	−2.15	.61				
	Environmental ward	.01 (.60)	.98	−1.17	1.19				

*Note.* Values in bold were found to be significant.

a LR test for adding random intercept χ^2^ = 85.23, *df* = 1, *p* < .01.

b LR test for adding random intercept χ^2^ = 4.22 *df* = 1. *p* > .05.

c Variance at time point 1 and 2.

**Table 4:** Mixed regression analysis for institutional factors associated with bullying (n = 220).

		Estimate (S.E)	Sig	95 % CI		Time 1	Sig	Time 2	Sig
PES Total^a^	Environmental ward pre	**7.6 (3.15)**	**.02**	**1.37**	**13.82**	278.61 (34.78)^d^	<.001	191.79 (31.46)^d^	<.0001
	Combined ward	−1.40 (2.93)	.63	−7.22	4.42				
	Social ward	−**7.03 (2.80)**	**.01**	−**12.59**	−**1.46**				
	Environmental ward	4.57 (3.15)	.15	−1.67	10.82				
Existence of hierarchy and order	Social ward pre	−**3.57 (1.44)**	**.01**	−**6.41**	−**.73**	33.43 (5.37)	<.001	18.20 (6.30)	.004
	Combined ward	−1.86 (1.60)	.25	−5.03	1.31				
	Social ward	−1.96 (1.74)	.26	−5.41	1.49				
	Environmental ward	.67 (1.50)	.65	−2.29	3.64				
Belief that bullying is inevitable^b^	Combined ward	.39 (.45)	.39	−.51	1.29	3.51 (.44)^d^	<.001	3.47 (.53)^d^	<.0001
	Social ward	−.46 (.44)	.29	−1.32	.4				
	Environmental ward	.59 (.43)	.17	−.26	1.43				
Absence of meaningful activities	Combined ward	−.34 (.48)	.49	−1.28	.61	3.14 (.49)	<.001	1.4 (.54)	.01
	Social ward	−**.92 (.46)**	**.046**	−**1.83**	−**.01**				
	Environmental ward	.2 (.45)	.45	−.69	1.09				
Raised social density^c^	Control ward post	−**.86 (.41)**	**.04**	−**1.68**	−**.04**	**7.04 (.88)**^d^	**<.001**	**4.90 (.74)**^d^	**<.001**
	Combined ward	−.23 (.60)	.71	−1.42	.97				
	Social ward	.64 (.58)	.27	−.52	1.80				
	Environmental ward	**2.63 (.57)**	**<.0001**	**1.49**	**3.78**				
Predictable Supervision	Combined ward	−.3 (.43)	.48	−1.15	.55	2.55 (.38)	<.0001	1.05 (.40)	.008
	Social ward	.06 (.41)	.88	−.75	.88				
	Environmental ward	.09 (.4)	.82	−.7	.89				

*Note.* Values in bold were found to be significant.

a LR test for adding random intercept χ^2^ = 4.27, *df* = 1. p < .05.

b Intercept was redundant.

c LR test for adding random intercept χ^2^ = 4.42, *df* = 1. p < .05.

d Variance at time point 1 and 2.

The final model showed an increase in the negative and blaming attitudes among the patients and staff members towards the victims of bullying as an effect of the combined intervention (Table 3). The LR test confirmed the significance of the negative effect (χ^2^ = 4.6, *df* = 1. <.05). However, across wards, there was a significant difference between the staff members and patients who held such attitudes (*Welch's F*(1,21.78) = 6.36, *p* = .02), with the former holding less of these attitudes. Meanwhile, for disapproval of bullying and support of victims, the final model showed a decrease for both combined *and* social interventions. The LR test confirmed the significance of the decrease^2^ in such attitudes (χ^2^ = 5.08 *df* = 1. *p* > .05 and χ^2^ = 7.43 *df* = 1. *p* > .01, respectively). However, the same model has also showed an increase in disapproval of bullying on the control ward where no interventions were implemented, which was confirmed with the LR test (χ^2^ = 4.47 *df* = 1. *p* > .05). There was no significant difference in degree of adoption of this attitude between patients and staff members on all four wards (*Welch's F*(1,31.38) = . 36 ns). The final model also demonstrated that the staff members and patients on the ward where social interventions were implemented had significantly lower victim protecting attitudes, compared to the control ward before changes were made. The LR test confirmed the significance of this difference at baseline (χ^2^ = 5.92, *df* = 1. *p* > .05).

The final model (Table 4) showed a positive effect of social intervention on the total score of the PES. The decrease in the total perception of the institutional factors associated with intra-group aggression was confirmed with the LR test (χ^2^ = 6.3, *df* = 1. *p* < .05). Furthermore, there was no difference in scores on this scale between patients and staff members on all four wards (*Welch's F*(1,30.57) = .29 ns). However, the same model showed that on the ward where changes to the physical environment were implemented, patients and staff perceived significantly more institutional factors supportive of bullying than those on the control ward before the intervention took place. The significance of this difference at the baseline was confirmed with the LR test (χ^2^ = 5.77, *df* = 1. *p* < .05). Similarly, the final model on presence of hierarchy and order showed that patients and staff members on the ward where social intervention was applied perceived the social system to be less rigid compared to those on the control ward before the changes were made. The LR test confirmed the significance of this baseline difference (χ^2^ = 6.18, *df* = 1. *p* > .05). Furthermore, the final model for meaningful activities showed a negative effect of social intervention. The significance of the decrease in perceived opportunities for fulfilling free time activities after the changes were made was confirmed with the LR test (χ^2^ = 3.97, *df* = 1. *p* < .05). There was no difference between the staff members and patients in this perception, *Welch's F*(1,26.98) = 2.28, *p* = .14. The final model for raised social density also showed a negative effect, albeit for the alterations in the physical environment, which was confirmed with the LR test (χ^2^ = 19.54, *df* = 1. *p* < .01) Moreover, this model showed that there was a decrease in perceived social density on the control ward where no interventions took place. The LR test confirmed this minor decline (χ^2^ = 4.45, *df* = 1. *p* < .05). Across four wards no significant difference between the patients and staff in this perception was found, *Welch's F*(1,34.93) = .20, *p* = .66.

## 7 Discussion

### 7.1 Study two

The intervention that included only alterations to the social climate exerted a positive effect on the factors associated with bullying. Alterations to the social environment resulted in a decrease in the institutional factors associated with intra-group aggression, but not a decrease in attitudes supportive of bullying. Thus, it would appear the impact was at an institutional and not a psychosocial level. However, similar social changes also facilitated a decrease in the attitudes that support victims and disapprove of bullying and added to the perception of there being no free time activities. This part of the results does not support expectations (Ireland et al., 2009). Meanwhile, making changes to the physical environment facilitated increases in social density, which is one of the institutional promoting factors for intra-group aggression. Last, and contrary to the MMBSS model, alterations to both physical and social environments increased blaming attitudes towards victims and decreased disapproval of bullies. Thus, the intervention impact on attitudes was more one of concern rather than benefit.

Taking into account the contradiction between the current results and previous research, it is possible that the predictions were disproved due to poor design and implementation at ward level. This explanation is supported by the decrease in disapproval of bullying on the control ward, where no alterations were made, and by the small number of changes present at the second time point. Nonetheless, there was also an unexpected but potentially relevant finding; there was only one significant difference in the attitudes towards bullying and perception of institutional factors between staff members and patients. This suggests that those spending time on the wards form a community that shares a psychosocial and physical environment, which equally affects and is formed by them. Targeting both staff and patients in interventions may therefore be key.

The quality of the intervention used is, however, a main limitation of the current study, especially given the disparity between the wards pre-intervention. This suggests an enhanced level of tailoring to wards is perhaps required. Equally, there is recognition of limitations in the analysis, accounting for the number of predictors, sample size and number of comparison groups. Whilst accepting the limitations on the inferential properties of the results, this was a low impact real-world preliminary intervention study in a highly specialist environment that was able to isolate a small number of positive findings for future research to further explore. Moreover, this study was able to indicate that interventions that directly changed the physical and social environment did not inhibit the predictors of intra-group aggression. It also suggests a larger replication study with more control over the implementation of intervention and an option to adjust changes to a given ward would be of benefit. Focusing on the positive findings that were isolated in relation to social climate in the current study, the ensuing study aims to focus on this aspect in particular. It will do so by trying to impact on positive change through a more targeted intervention approach that focuses on the role of social community.

## 8 Materials and methods

### 8.1 Study three

The current study evaluates a means of inhibiting bullying related factors not by altering the physical and social environment independently, as was attempted in Study 2, but more indirectly by engaging patients in meaningful activities to enhance the social component of their environment. Furthermore, since participating in meaningful group-based activities can be seen as meeting an individual's needs via a community (Chavis et al., 2008), the intervention aimed to increase a positive sense of community, a factor not fully captured in the earlier study. Community is a salient aspect of the social context and according to the MMBSS absence of a positive community could promote inter-group aggression. Consequently, study 3 evaluates whether providing patients with *group-based* or *individual* meaningful activities can improve their sense of community and decrease the institutional factors associated with intra-group aggression, as compared to a control group. Again, the specific wards chosen were selected by hospital management and not the researchers. All patients on the identified wards were able to engage.

In order to maximise the positive effect of the implemented activities, their nature was determined during interviews and focus groups with patients (Cleary et al., 2013), to ensure buy in and their meaningful nature. For instance, for the group-based activities patients asked for pool tournaments, movie nights and ward football games. Meanwhile, the individual meaningful activity reflected any activity that was available to the participant at that time in the secure hospital and that they enjoyed doing.

#### 8.1.1 Participants

There were three components to the final sample;

a.) *Baseline patient sample* (n = 57 male patients) to establish the predictors of bullying and victimisation. This sample presented with an age range of 20–62 (with most, 71.4%, aged between 31 and 51). Eighty-one per cent of patients identified as White. Sixty-seven per cent of the sample reported their diagnoses, under the MHA, as mental illness, 19% as psychopathic disorder and 9% reported having combined diagnoses. Five per cent of the sample did not disclose their diagnosis.
b.) *Patient baseline and post sample*, including the baseline sample and those who agreed to participate in the second round of data collection and completed questionnaires, which took place six months after the intervention commenced. Of the 57 baseline patients, 24 received group interventions, 20 individual intervention and 13 none. Twenty-seven agreed to participate in the follow up, thereby decreasing group sizes to: 13, nine, and five respectively.
c.) *Patient baseline, patient post sample and staff sample* who agreed to participate in both rounds of data collection. This comprised 307 baseline participants (53 patient and 254 staff members) who took part in the evaluations before the interventions commenced. A hundred and twenty-one of these participants (23 patients and 98 staff) were in the group intervention condition, 111 (17 patients and 94 staff) the individual intervention condition, and 75 (13 patients and 62 staff) were in the control group. However, only 107 participants (25 patients and 82 staff) took part in the post intervention data gathering, which took place six months later. From these, 50 participants (12 patients and 38 staff) received group intervention, 44 (eight staff and 36 staff) received individual intervention, and 13 (five patients and eight staff) served as control.

#### 8.1.2 Evaluation measures

All participants consented to complete the measures, with ethical approval again obtained from the University of Central Lancashire Ethics Board for Psychology, with regulations complied with. The measures comprised the Patient Bullying Scale (PBS), Prison Environment Scale (PES), and Direct and Indirect Patient behaviour Checklist-Hospital version Revised (DIPC-HR). These are outlined in Study 1. The DIPC-HR was again only given to patients. In addition, the following measures were employed:

*Sense of Community Index 2 (SCI-2:* Chavis et al., 2008*),* a 12 item Likert Scale to evaluate individual sense of belonging to a community. It comprises four subscales; perceiving oneself as a member of a community; feeling that one has the ability to influence a community; meeting individual psychological needs via a community; and experiencing an emotional connection with a community. Example items include, ‘I feel hopeful about the future of this community’ and ‘I can trust people in this community’. The total index and its subscales have been shown to have good reliability (α’s ranging from .79 to .94: Chavis et al., 2008). Patients and staff completed this measure.

*Negative Psychological Sense of Community (NPSOC:* Mannarini et al., 2014*),* a 32 item Likert measure to estimate the factors that drive an individual's disengagement from a community. It includes four components; perceiving oneself as distinct from a community; feeling alienated; feeling frustration with a community; and abstention from engaging in community activities. Example items include: ‘I feel I'm different from the members of this community’ and ‘I am not in tune with the lifestyle of this community’. The total NPSOC and its subscales have been shown to have good reliability (α’s ranging .78 to .95: Mannarini et al., 2014). Only patients completed this measure.

## 9 Results

### 9.1 Study three

A correlational analysis was initially employed to identify the constructs that were relating to intra-group aggression, accounting for the novel use of the community. Spearman rank order correlations were run between the DIPC-HR bullying and victimisation totals, the NPSOC and SCI-2 (n = 57). Membership in a community had a weak positive association with self-reported bullying (*r* = .27, *p* < .04), with no further significant correlations with bullying noted (all *r*'s ≤ = .17 ns). Victimisation reports presented with moderate positive associations with the total negative sense of community (*r* = .30, *p* < .03), feeling alienated from a community (*r* = .30, *p* < .02), viewing yourself as different from a community (*r* = .30, *p* < .02) and feeling frustrated with the community (*r* = .25, *p* < .007). There were no further significant correlations with victimisation (all *r*'s ≤ = .17 ns).

Separate binominal regressions were then employed using the positive and negative sense of community, to test whether the constructs highlighted in the previous step could predict involvement in patient bullying/victimisation. The predictors are presented in Table 5.

**Table 5:** Predictors for bullying and victimisation (n = 57).

Category	Predictor	Exp. B	S.E.	Sign	Model Chi square	Hosmer Lemeshow test	Nagelkerke R^2^	% Correctly classified	2 Log Likeli-hood
Bullying	SCI Membership	1.06	.06	.3	15.36	.03	.03	63.2	73.84
Victimisation	NPSOC Total Score	**1.02**	**.01**	**.04**	**5.51**	**.60**	**.11**	**68.4**	**73.67**
	NPOSC Frustration	**1.09**	**.04**	**.02**	**5.02**	**.66**	**.15**	**66.7**	**71.92**
	NPSOC Alienage	1.06	.03	.09	5.44	.49	.07	59.6	75.41
	NPSOC Distinction	**1.09**	**.04**	**.02**	**4.74**	**.79**	**.14**	**64.9**	**72.12**
	SCI Membership	1.04	.05	.41	10.84	.15	.02	52.6	77.89

*Note*. Results in bold were found to be significant at .05 level.

Predictors that were found significant were also subjected to a ROC curve analysis to verify the correctness of the classification. For total negative sense of community AUC was .66 (SE = .07, Asymptotic significance = .04, CI = .52–.81), suggesting a poor discriminatory ability. Similarly, for seeing oneself as distinct from a community AUC had a value of .69 (SE = .07, Asymptotic significance = .02, CI = .55–.83) indicating poor to moderate discriminatory power. Meanwhile, for feeling frustrated due to inability to fulfil one's needs via a community, AUC was moderate with value of .70, (SE = .07, Asymptotic significance = .01, CI = .57–.84).

Due to high attrition rates and potential variance (as well as within participants) mixed regressions were then used to assess whether the intervention had an effect on the perception of the institutional factors and sense of community. This was used for measures that were completed only by the patients as well as for those completed by staff members *and* patients. Initially, for each outcome variable, a repeated measures model with an unspecified 2 × 2 covariance matrix for an individual effect at each time point was created, then a random within subject effect was added to the model (the Likelihood ratio test with *df* = 1 was used to determine fit of the new model). The predictors were time (before the intervention and six months after), group (dummy variables were created for individual and group-based activities, while the control group was used as a reference point) and the interactions of each group of the participants with time. The difference between the groups at the pre-intervention time and the difference within the control group with the passing of time were removed when the differences between the models were not significant. The final model with significant interactions coefficients was also tested against the model without interactions. Similar to the second study, a Welch test was used to assess whether there were differences between the ratings of staff members and patients on the scales that had significant changes six months after the implementation of the intervention. In order to identify the predictors of engaging in bullying and experiencing victimisation among those aspects of the positive and negative sense of community that are related to intra-group aggression, separate binominal regressions were run.

The following mixed effect regression model was used for each outcome score Y as obtained at the time *t* for an individual *i:*

Y_ti_ = β_0_ + β_1_Post + β_2_Indiviudal_Intervention + β_3_Group_Intervention + β_4_Post*Individual_Intervention + β_5_PrePost*Group_Intervention + *u*_*i*_ + *e*_*ti*_

where t = 0 or 1 for baseline and six months after respectively and post = 1 if t = 1,

*u*_*i*_ is a random effect of an individual *i*, and *e*_*ti*_ is a random effect of an individual *i* at the time point *t* also referred to as residual variance per individual per time point.
β_0_ - the outcome mean at the baseline in comparison group.
β_1_ – the mean change from baseline to six months afterwards within the comparison group.
β_2_ - the mean baseline difference between individual interventions group and comparison group.
β_3_ - the mean baseline difference between group interventions group and comparison group.
β_4_ - the difference in mean change from baseline to six months afterwards between individual interventions group and comparison group, which is also the difference at six months afterwards, as there is no difference at baseline.
β_5_ – the difference in mean change from baseline to six months afterwards between group interventions group and comparison group, which is also the difference at post-test, as there is no difference at baseline.

The results for institutional (PES) factors) are presented in Table 6 and for community factors (NPSOC and SCI-2) in Table 7.

**Table 6:** Mixed regression analysis for institutional (PES) factors associated with bullying (n = 414, includes both staff and patients).

		B (S.E.)	Sig	CI		WS	Sig	BS	Sig
PES total score	Individual intervention baseline	−**6.36 (1.73)**	**<.001**	**−9.77**	**−2.96**	192.49 (23.25)	<.001	24.85 (20.22)	.22
	Group intervention post	**−5.37 (2.26)**	**.02**	**−9.83**	**−.92**				
	Individual intervention post	2.83 (2.54)	.27	−2.17	7.83				
Existence of hierarchy and order	Individual intervention baseline	−**3.01 (.88)**	**.001**	−**4.74**	−**1.29**	46.61 (5.95)	<.001	9.3 (5.48)	.09
	Group intervention post	−**2.48 (1.13)**	**.03**	−**4.71**	−**.25**				
	Individual intervention post	1.01 (1.26)	.42	−1.48	3.50				
Belief that bullying is inevitable	Individual intervention baseline	−**.55 (.27)**	**.045**	−**1.08**	−**.01**	4.37 (60)	<.001	.98 (56)	.09
	Group intervention post	−.23 (.35)	.51	−.92	.45				
	Individual intervention post	.74 (.39)	.06	−.02	1.51				
Absence of meaningful activities	Individual intervention baseline	−**.99 (.27)**	**<.001**	−**1.52**	−**.46**	4.93 (.61)	<.001	.25 (51)	.63
	Group intervention post	−**1.46 (.36)**	**<.001**	−**2.16**	−**.76**				
	Individual intervention post	.56 (.4)	.16	−.23	1.35				
Raised social density	Group intervention post	**.62 (.31)**	**.046**	**.01**	**1.23**	5.74 (.46)	<.001	3.97 (.55)	<.0001
	Individual intervention post	.37 (.32)	.26	−.28	1.01				
Predictable Supervision	Group intervention post	−.21 (.25)	.39	−.69	.27	2.48 (.30)	<.001	.19 (.25)	.46
	Individual intervention post	−**.63 (.26)**	**.02**	−**1.14**	−**.12**				

*Note*. Results in bold were significant.

**Table 7:** Mixed regression analysis for negative sense of community (n = 84, includes only patients).

		B (S.E.)	Sig	CI		WS	Sig	BS	Sig
NPSOC	Group intervention post	−12.39 (8.41)	.15	−29.59	4.81	587.43 (211.02)	.005	465.51 (279.20)	.10
	Individual intervention post	−17.18 (9.98)	.10	−37.54	3.18				
NPSOC Frustration	Group intervention post	−2.61 (2.33)	.27	−7.34	2.12	46.66 (15.61)	.003	28.42 (18.87)	.13
	Individual intervention post	−4.90 (2.76)	.08	−10.39	.70				
NPSOC Alienage	Group intervention post	−**4.99 (2.23)**	**.03**	−**9.54**	−**.45**	41.56 (14.65)	.005	30.82 (18.93)	.10
	Individual intervention post	−5.02 (2.64)	.07	−10.40	.36				
NPSOC Abstention	Group intervention post	−2.88 (1.95)	.15	−6.89	1.12	29.58 (9.66)	.002	41.86 (15.95)	.009
	Individual intervention post	−3.97 (2.33)	.10	−8.73	.79				
NPSOC Distinctiveness	Group intervention post	−2.24 (2.40)	.36	−7.13	2.66	52.81 (21.37)	.01	18.91	.43
	Individual intervention post	−3.35 (2.84)	.25	−9.11	2.41				

*Note*. Results in bold were found to be significant.

The final model for the absence of meaningful activities showed a positive effect (Table 6). The significance of the decrease in those who held this view was confirmed with the LR test (χ^2^ = 16.43, *df* = 1 *p* < .01). Positive effect was also present for the individual variation of the intervention, as the significance of the improvement in the perceived variety of free time activities was confirmed via the LR test (χ^2^ = 16.43, *df* = 1 *p* < .01). Staff members and patients did not differ significantly in this respect (*Welch's F*(1,32.47) = 2.45, *p* = .13). As the intervention was the introduction of the meaningful activities, these results serve as a manipulation (adherence) check for the quasi-experimental conditions.

The final models for total score on the PES, presence of hierarchy and order, perception of bullying as inevitable, and for absence of meaningful activities, showed that the patients and staff, who would later undergo individual interventions, reported all these aspects to be lower compared to the control group. The significance of these differences at baseline was confirmed with the LR tests (χ^2^ = 13.39, *df* = 1 *p* < .01; χ^2^ = 11.71, *df* = 1 *p* < .01; χ^2^ = 4.05, df = 1 *p* < .05; χ^2^ = 16.43, *df* = 1 *p* < .001, respectively). However, the following intervention effects were also found; final model for total score on the PES showed a positive effect of the group-based variation of the intervention, which was confirmed with the LR test (χ^2^ = 5.64, *df* = 1 *p* < .05). Furthermore, there was no difference between staff members and patients in this regard (*Welch's F*(1,32.27) = .004, *p* = .95). Similarly, the final model for the presence of hierarchy and order showed a positive effect of the group-based variation of the intervention. The significance of the decrease in rigidity of social structure was confirmed with the LR test (χ^2^ = 4.8, *df* = 1 *p* < .05). The patients and staff members also did not differ in this perception (*Welch's F*(1,29.59) = .18, *p* = .68).

Nevertheless, the final model for raised social density showed a negative effect of the group-based variation of the intervention. The LR test confirmed the significance of the increase in this perception (χ^2^ = 4.05 *df* = 1 *p* < .05). Staff members and patients did not differ significantly in their evaluation of social density (*Welch's test, F*(1,36.63) = .57, *p* = .46). Lastly, the final model for predictable supervision revealed a positive effect of the individual variation of the intervention. The significance of the decrease in the perception that supervision is predictable was confirmed with the LR test (χ^2^ = 5.87 *df* = 1 *p* < .05) and again there was no difference between staff and patients (*Welch's F*(1,38.19) = .48, *p* = .49).

The final model for feeling alienated from a community showed only one positive effect of the group-based variation of the intervention (Table 7). The significance of the decrease in the alienation six months after the intervention was confirmed with the LR test (χ^2^ = 4.45 *df* = 1 *p* < .05).

## 10 Discussion and conclusions

This series of preliminary studies produced mixed results with regards to what predicted intra-group aggression in secure psychiatric settings and the potential impact of intervention on these factors. It is, nevertheless, a novel set of studies in that despite high rates of bullying and victimisation reported in secure settings (e.g. Ireland and Bescoby, 2005; Ireland and Rowley, 2007; Cooper et al., 2011; Haufle & Wolter, 2015), there remains a lack of attention given to designing, implementing and evaluating interventions (Ireland, 2012). The current study aimed to begin to address this issue by providing an empirical base for starting to consider the design and implementation of interventions that could reduce bullying in secure hospitals.

As a whole, the research demonstrated that interventions targeting bullying factors *indirectly* appear to produce more positive effects than a direct targeting of factors. For example, making direct alterations to the physical and social environment facilitated only one positive effect, namely it reduced the institutional factors associated with bullying and victimisation. However, it also generated an unintended consequence in the form of an increase in the attitudes and cognitions supportive of intra-group aggression. Thus, although the research found that there were physical and social environmental factors associated with intra-group aggression, thereby supporting the respective predictions, impacting positively on these factors was less clearly indicated. The physical and social environmental factors also appeared very specific, with factors such as hierarchy and order, and raised social density, particularly important, alongside unhelpful attitudes. The inevitability of bullying was a salient example of the latter.

However, it was the promotion of positive change to the aggression-enhancing aspects of the physical and social environment that appeared particularly difficult to address. Although making changes to the social climate was having some impact, making changes more broadly to the physical and social environment was not having an appreciable impact. Thus, the predictions that making positive changes to the aggression-enhancing aspects of the physical and/or social environment would reduce the factors associated with intra-group aggression was not broadly supported, or the prediction that making changes to both (physical and social) simultaneously would have an accumulatively more positive impact.

What did, nonetheless, emerge as important was a subtler aspect of the social environment, namely the community. This has been indicated as an important consideration in understanding and managing secure based bullying, both at a theoretical (e.g. MMBSS: Ireland, 2012) and empirical (e.g. Ros et al., 2013) level. Enhancing the community did appear to have a positive impact on some discrete factors associated with increasing intra-group aggression. However, it was not achieved through direct action but appeared more as a product of other factors. Put simply, the sense of community was arguably increased by the introduction of meaningful activities for patients. Such activities had positive effects. They were associated with improvements in bullying-related cognitions and appeared to ameliorate some aspects of a negative sense of community. Specifically, meaningful activity intervention appeared associated with decreases in the belief that bullying was inevitable and in the perceived rigidity of social hierarchy. Both of these factors, as indicated earlier, were discretely predictive of engaging in bullying, with the latter also found to be predictive of victimisation.

The findings suggest that encouraging the engagement of patients in supervised group activities reduces the likelihood that they will be involved in intra-group aggression; in short, engaging patients in meaningful activity serves to have a potential by-product (indirect) effect of reducing the factors associated with bullying. It could be speculated that it achieves this goal via two means; by enhancing a positive sense of community through the group focus and/or by occupying time and reducing boredom, with boredom known to aggravate involvement in bullying (Ireland, 2012). Experiencing victimisation was also characterised, not only by the presence of institutional factors, but also by an increased negative sense of community. These findings converge with the basic propositions of the MMBSS model and also suggest some potential adjustments that could be made.

Overall, it was demonstrated that intra-group aggression is a function of certain social environment and individual factors. An increase in the presence of factors, such as social dominance hierarchies and adoption of the attitude that bullying is inevitable, raise the likelihood that a patient will engage in bullying. This replicated previous findings among prisoners (Allison and Ireland, 2010). At the same time, those patients who held the belief that bullying can have positive connotations were also more likely to engage in intra-group aggression. This finding extended the previous study of Cooper et al. (2011), by showing that beliefs similar to a machismo cognitive style could predict engagement in aggressive behaviour towards other patients.

Consistent with the desensitisation pathway of the MMBSS (Ireland, 2012), where normalisation of aggression supportive cognitions paves the way to enacting aggression, the current findings demonstrate how beliefs and attitudes supportive of bullying can predict engagement. Beliefs therefore appear key elements and yet they also represent perhaps the most challenging aspects to change. There are, nevertheless, worthy of raised attention in the MMBSS model and perhaps should be considered a primary feature with regards to perpetration. The current studies further suggest that the ‘environment and prior characteristic pathway’ of the MMBSS is represented more by the predictors of victimisation. Experiencing raised social density, which is a part of the physical environment, increased the likelihood of suffering from intra-group aggression by 36%. Moreover, experiencing a negative sense of community (the individual characteristic that reflects social standing) increased the chance of victimisation. Connection with a community appeared particularly important therefore in terms of victimisation as opposed to perpetration. It also extends the results of Sekol (2016) who found that lack of peer support was predictive of victimisation among children in care homes, suggesting there is something positive about having support and being part of a community, perhaps as a preventative approach. Furthermore, negative attitudes/beliefs did not predict victimisation, further highlighting the importance of other factors.

The similarity evidenced in the current research between the ‘environment and prior characteristics pathway’ of the MMBSS and predictors of victimisation suggests that it might not only be a pathway to enacting intra-group aggression, but also be a pathway resulting in victimisation, whereas the ‘desensitisation pathway’ may be associated more with explaining perpetration. The MMBSS currently makes no such distinction in terms of a victimisation and/or perpetration preferred pathway, but the collective results of the current research suggests it is worthy of further consideration and refinement.

The current studies are not without their limitations, however, some of which have been captured earlier. The research is preliminary and limited by reduced sample size and attrition rates, both of which impact on the choice of analysis and interpretation of the same. There was also no assessment of reading ability. The current study also did not capture women. There is a comparative absence of women in high secure psychiatric care after such services were significantly downsized in the UK from 2003 onwards, with a preference now for enhanced medium secure services for women. Consequently, the results cannot be applied to the experiences of women detained in enhanced levels of security in psychiatric settings. In addition, the study focuses on the experiences within the same hospital; although this has the benefit of allowing for interventions to be applied to the same environment, it also questions how generalisable the findings are beyond this setting. There was also overlap between the chosen wards and populations across the distinct studies. Nevertheless, there was a considerable time delay between each study, thus arguably removing issues such as practice effects or prior intervention influence. It is also important to acknowledge how specialist this high secure male population is and the real-world application of intervention. It was not possible, for example, to determine the level of adherence to intervention or to ascribe a quantitative figure to the changes made (such as a percentage change) other than ensuring that changes were in place and checking this through monthly meetings with the ward management team. However, the aim of this research is not just to present findings but also to highlight the challenges in conducting research of this nature. An increased focus on the implementation of intervention and adherence to the same would have undoubtedly been valuable, even notwithstanding the challenges in ensuring that this takes place. This does represent a sensitive area of study; an under-reporting of bullying and victimisation and general difficulties on wards is not unexpected but future research could perhaps supplement the current approach by also collecting objective data on aggression (e.g. staff reports).

There are perhaps three main contributions of these preliminary studies. They partly confirm the desensitisation pathway of the MMBSS for an adult male psychiatric sample detained in high secure conditions, further highlighting how such a pathway may be most valuable to describing the predictors of perpetration. They also highlight the relevance of the social climate and how this is a complex concept worthy of addressing through a variety of means, which can include indirect means. It also represents the first attempt at empirically evaluating a theoretically informed anti-bullying intervention among a forensic population, which has sought to make direct and indirect alterations to the environment. As a result, it highlights the challenges and areas where there is a need for improvement, whilst also promoting the notion that some by-products of aggression intervention can serve to unexpectedly promote aggression whilst others may unexpectedly result in benefits, such as the indirect benefits of engagement in group based meaningful activity. The current studies remain exploratory but they do perhaps provide a basis for future intervention work that captures both empirical findings as a basis for delivery and increased attention to implementation.

## Declarations

### Author contribution statement

J. L. Ireland: Conceived and designed the experiments; Performed the experiments; Analyzed and interpreted the data; Wrote the paper.

I. Sebalo: Analyzed and interpreted the data; Wrote the paper.

K. McNeill, K. Murphy, M. Lewis, L. Greenwood, T. Nally: Performed the experiments; Analyzed and interpreted the data.

G. Brewer: Analyzed and interpreted the data.

C.A. Ireland, S. Chu: Conceived and designed the experiments; Performed the experiments.

### Funding statement

This research did not receive any specific grant from funding agencies in the public, commercial, or not-for-profit sectors.

### Competing interest statement

The authors declare no conflict of interest.

### Additional information

No additional information is available for this paper.

## Footnotes

1 Spearmans was used as the assumptions of Pearson were violated; Spearmans is thus more appropriate.

2 This subscale is reverse scored.
